# Supplementary material for: Content-rich biological network constructed by mining PubMed abstracts
Source: BMC Bioinformatics. 2004 Oct 8;5:147. doi: 10.1186/1471-2105-5-147 (PMC528731; doi:10.1186/1471-2105-5-147)
Supplement: Additional File 2 — The original results of the above study (non-essential files are deleted to keep the file size under the limit set by BMC bioinformatics). [file 1471-2105-5-147-S2.bz2 › chilibotAdditionalFile2/dip20/95ID9178909E402/html/PIK3R1_CBL.html]

 


 **PIK3R1** and **CBL** 
  
Found 24 abstracts in PubMed, retrieved 20.  
 

 What does Google say? 
 PDF only 
| .edu only 

---

**Interactive relationship** (e.g. stimulation, inhibition, etc)

**Neutral relationship**- The adaptor protein SETA CIN85 Ruk is involved in regulating diverse signal transduction pathways, including the internalization of tyrosine kinase receptors via the  **Cbl**  ubiquitin ligases, and attenuating PI3K  [ **PIK3R1** ]  activity by interaction with its regulatory subunit.  Ref: 12771190 J Cell Sci< MedlineTA>J Cell Sci,
- The p85 regulatory subunit of phosphatidylinositol 3 kinase PI3K  [ **PIK3R1** ]  was identified as a substrate for  **Cbl**  b.  Ref: 11526404 Nat Immunol, 2001
- The docking proteins that the BCR uses to recruit PI3K  [ **PIK3R1** ]  include CD19,  **Cbl** , Gab1, and perhaps Gab2.  Ref: 11043767 Immunol Rev, 2000
- This process has been proposed to be mediated by an interaction between the Src family kinase LYN and the p85 subunit of PI3K  [ **PIK3R1** ]  and or through p85 membrane recruitment mediated by  **CBL**  and or CD19.  Ref: 10551821 J Biol Chem, 1999
- Our findings suggest that  **Cbl**  participates in mediating effects of rosiglitazone on PI3K  [ **PIK3R1** ] , PDK 1, and PKC lambda and the glucose transport system.  Ref: 11956152 Endocrinology, 2002
- **Cbl** , IRS 1, and IRS 2 mediate effects of rosiglitazone on PI3K  [ **PIK3R1** ] , PKC lambda, and glucose transport in 3T3 L1 adipocytes.  Ref: 11956152 Endocrinology, 2002
- this  **Cbl**  dependent pathway complements the IRS 1 and IRS 2 pathways for activating PI3K  [ **PIK3R1** ] , PDK 1, and PKC lambda during combined actions of rosiglitazone and insulin in 3T3 L1 cells.  Ref: 11956152 Endocrinology, 2002
- Whereas CD19 does not appear to be involved in this SYK dependent pathway, the SYK substrate  **CBL**  is likely involved as the dominant negative SYK markedly attenuates  **CBL**  tyrosine phosphorylation and completely blocks the BCR dependent association of  **CBL**  with p85 PI3K  [ **PIK3R1** ] .  Ref: 10551821 J Biol Chem, 1999
- Under our conditions, hydrogen peroxide induced PI3K  [ **PIK3R1** ]  and Akt activation was independent of Lyn, Syk,  **Cbl** , BCAP, or Ras when each was eliminated individually either by mutation or by a specific inhibitor.  Ref: 12627965 Biochemistry, 2003
- The enhanced activation of  **Cbl**  b T cells was suppressed by the inhibition of PI3K  [ **PIK3R1** ] .  Ref: 11526404 Nat Immunol, 2001

**Non-interactive relationship** (e.g. studied together, co-existance, homology, etc.)

- Proteolysis independent regulation of PI3K  [ **PIK3R1** ]  by  **Cbl**  b mediated ubiquitination in T cells.  Ref: 11526404 Nat Immunol, 2001
- The enhanced Akt activity induced by stimulation of endothelial CD40 was temporarily correlated with the association of CD40 with TRAF6, c  **Cbl** , and the p85 subunit of PI3K  [ **PIK3R1** ] .  Ref: 12637493 J Biol Chem, 2003
- About 50% of the T cells from young mice that formed conjugates with peptide pulsed APC were found to form complexes, at the site of binding to the APC, containing CD3epsilon, linker for activation of T cells LAT, and Zap 70 in a central area and c  **Cbl** , p95 vav, Grb  [ **PIK3R1** ]  2, PLC gamma, Fyn, and Lck distributed more uniformly across the interface area.  Ref: 11207267 J Immunol, 2001
- 70Z  **Cbl**  retains the ability to enhance NFAT and activating protein 1 AP1 activity in the absence of Crk L p85 PI3K  [ **PIK3R1** ]  association.  Ref: 9988765 J Biol Chem, 1999
- accompanied by increases in tyrosine phosphorylation of  **Cbl**  and  **Cbl**  dependent increases in PI3K  [ **PIK3R1** ]  and PKC lambda activity.  Ref: 11956152 Endocrinology, 2002
- Expression of negative dominant Akt inhibited the activation of endogenous Akt through CD40 stimulation, despite the observation that association of CD40 with TRAF6, c  **Cbl** , and PI3K  [ **PIK3R1** ]  was intact.  Ref: 12637493 J Biol Chem, 2003
- Utilizing unique phospho specific antibodies generated against various tyrosines of c Met, we show that Y1003 binding site for c  **Cbl**  and a negative regulatory site, Y1313 binding site for PI3K  [ **PIK3R1** ] , Y1230 Y1234 Y1235 autophosphorylation site, Y1349 binding site for Grb2, Y1365 important in cell morphogenesis are phosphorylated in response to HGF 40 ng ml, 7.5 min in H69 cells.  Ref: 12611639 J Cell Mol Med< MedlineTA>J Cell Mol Med, 2003
- We demonstrate that 70Z  **Cbl**  shows increased basal and activation induced tyrosine phosphorylation and association with Crk L and p85 PI3K  [ **PIK3R1** ]  in Jurkat T cells.  Ref: 9988765 J Biol Chem, 1999
- This was associated with phosphorylation of the adapter protein p120  **Cbl**  and an increase in  **Cbl**  associated PI3K  [ **PIK3R1** ]  activity.  Ref: 11123288 J Immunol, 2001
- We conclude that 70Z  **Cbl**  induced NFAT AP1 activation requires the phosphotyrosine binding domain but not Crk L p85 PI3K  [ **PIK3R1** ]  association.  Ref: 9988765 J Biol Chem, 1999
